# Integrative Proposals of Sports Monitoring: Subjective Outperforms Objective Monitoring

**DOI:** 10.1186/s40798-022-00432-z

**Published:** 2022-03-26

**Authors:** Lluc Montull, Agne Slapšinskaitė-Dackevičienė, John Kiely, Robert Hristovski, Natàlia Balagué

**Affiliations:** 1 Complex Systems in Sport Research Group, Institut Nacional d’Educació Física de Catalunya (INEFC), Universitat de Barcelona, Barcelona, Spain; 2 University School of Health and Sport, University of Girona, Girona, Spain; 3 Department of Sports Medicine, Faculty of Nursing and Faculty of Public Health, Health Research Institute, Medical Academy, Lithuanian University of Health Sciences, Kaunas, Lithuania; 4 Institute of Coaching and Performance, School of Sport and Wellbeing, University of Central Lancashire, Preston, PR1 2HE UK; 5 Complex Systems in Sport Research Group, Faculty of Physical Education, Sport and Health, Ss. Cyril and Methodius University in Skopje, Skopje, Macedonia

**Keywords:** Complex adaptive systems, Self-regulation, Technology, Awareness, Health, Performance

## Abstract

Current trends in sports monitoring are characterized by the massive collection of tech-based biomechanical, physiological and performance data, integrated through mathematical algorithms. However, the application of algorithms, predicated on mechanistic assumptions of how athletes operate, cannot capture, assess and adequately promote athletes’ health and performance. The objective of this paper is to reorient the current integrative proposals of sports monitoring by re-conceptualizing athletes as complex adaptive systems (CAS). CAS contain higher-order perceptual units that provide continuous and multilevel integrated information about performer–environment interactions. Such integrative properties offer exceptional possibilities of subjective monitoring for outperforming any objective monitoring system. Future research should investigate how to enhance this human potential to contribute further to athletes’ health and performance. This line of argument is not intended to advocate for the elimination of objective assessments, but to highlight the integrative possibilities of subjective monitoring.

## Key Points

- There is a disconnect between the complexity underpinning human health and performance and the deterministic models through which athletic monitoring and assessment are conceptualized.
- While focusing on collecting and processing large amounts of data, analysts, scientists and coaches may forget the outstanding potential of the human neurobiological system to dynamically, and rapidly, integrate massive amounts of personal and environmental information.
- Understanding and valuing subjective-based monitoring are crucial to enhance athletes’ awareness and promote their self-consciousness, autonomy and self-regulation of health and performance.

## Introduction

In sporting contexts, the use of monitoring tools and physical activity trackers providing training and general health data has recently expanded dramatically. In 1954, hand-timing regular training runs was considered unusual [1]. However, as technology, beginning in the early 1980s, began to rapidly evolve, new possibilities to assess internal and external training loads during training and competition became available [1]. Today, new wireless technologies are expanding to provide simultaneous data related to biomechanical, physiological and performance variables [2–5].

Compiling and integrating multiple internal and external variables through a variety of mathematical algorithms (e.g., ACWR) [6–8] and making predictions on the basis of artificial intelligence software [9, 10] seem to be leading current, and future, steps in sports monitoring. In effect, there appears to be a tacit assumption that the collection of objective ‘big data’ is the key to provide more pertinent and relevant information to promote athletes’ health and improve athletic performance [11]. Further, moves towards cyborgization—the integration of measurement and computational technologies into human bodies [12, 13]—suggest that current trends of sports monitoring are continuing to evolve towards the tracking of ever-increasing streams of objective data.

Accordingly, coaches and sports scientists are motivated to collect a growing diversity of data, using an array of commercially available assessment technologies [14]. Professional sport organizations are also investing heavily (in terms of time, money and specialized human resources) in new technologies. Providing previously unavailable metrics (e.g., acceleration) without any clear vision of how the new information will be interpreted and actioned appears to be the rule [14]. This approach, however, is not well aligned to the scientific ideal of developing models and hypotheses and then testing deductively. Instead, there is a commercial drive to develop new technologies in advance of hypotheses, to market these technologies based on non-existent or insufficient evidence and to encourage practitioners to embark on little more than 'fishing expeditions' to see what new technologies may offer [15].

The expansive range of emerging technologies may leave analysts and sports science practitioners struggling to deal with collecting, processing and interpreting large quantities of objective data, as occurs, for example, in elite soccer contexts [16, 17]. This, potentially excessive, focuses on objective data may serve to distract practitioners from the outstanding potential of humans to innately integrate and process multiple streams of highly sophisticated information at high speed [18]. Although objective data may be helpful in some contexts (e.g., position data of teams, athletes’ physiological response) [19, 20], it may not inevitably lead to greater insight and better understanding of health and performance [21]. Importantly, there is a clear and problematic disconnect between the neurobiological complexity underpinning human performance and the mechanistic, deterministic, albeit complicated and intricate, biomedical model through which we conventionally conceptualize both athletic training paradigms and athletic assessment protocols [22–24].

This apparent disconnect highlights an evident need to evaluate the advantages and costs of technology-enabled objective information against those of athlete-generated subjective information for continuously integrating multilevel, multisource and multimodal information. Accordingly, the objective of this paper is to reorient the current integrative proposals of sports monitoring by re-conceptualizing athletes as CAS.

## Monitoring Complex Adaptive Systems

Contemporary athletic monitoring philosophies perpetuate a mechanistic and reductionist philosophical stance in sports training that envisions athletes and teams as linear, deterministic systems composed by multiple components. This view, founded in an historically pervasive, but scientifically inaccurate conception, guides coach decision-making processes in relation to athletes’ health prevention and performance [7, 25].

Neurobiological systems are seen as dominated by components that establish inherently linear, deterministic cause-and-effect relationships among them. Such deterministic systems, when perturbed, respond to imposed stimuli with proportional and predictable adaptive responses as an externally predesigned product of their component behaviours. Yet, across the biological and neurological sciences, this conventional biomedical interpretation has been overthrown by the overwhelming evidence illustrating that human neurobiology is more appropriately conceptualized as a CAS: a nonlinear and dynamic system comprised of multiple embedded complex sub-systems which collaboratively and collectively share co-modulating information both vertically (e.g., genes, cells, tissues, organs, etc.) and horizontally (e.g., among molecules, cells, organs, etc.) to support the continued survival of the unified whole [22, 26].

Sport behaviours, accordingly, are guided and constrained by an embedded and embodied, experience-dependent and goal-directed performer–environment interactions. Hence, athletes’ self-monitoring and self-regulation are important competencies of biological intelligence to perform in cooperative–competitive environments [27].

The failings of applying traditional linear logic to complex sport phenomena have been previously reported, such as in the case of sport injuries [14, 28–31]. For example, the critical tensile forces that produce muscle rupture in vitro cannot be directly transferred to the complex muscle contraction in vivo [23, 32]. Over the past two decades, the science of complex systems, and particularly the nonlinear dynamic systems theory, has begun to percolate into various branches of sports science [22, 23, 33–35]. Recently, the network physiology of exercise—a framework studying the nested dynamics of vertical and horizontal physiological network interactions to understand how physiological states and functions emerge—has been introduced to exercise [22, 26]. This complex system-based approach appears more capable of eloquently capturing the theoretically and methodologically sport-related phenomena such as injuries [29, 30], fatigue [36, 37] or motor control and learning [38–40].

Tables 1 and 2 contrast traditional and complex system-based characteristics of athletes and training process, respectively.

**Table 1:** Characteristics of athletes from traditional and complex system-based approaches. Adapted from Pol et al. [23], with permission

Approach	Traditional	Complex system-based
Concept of organism	Machine	CAS
Control	Internal/external programmes	Synergies^1^
Organization	Centrally regulated	Self-organized^2^
Interaction with the environment	Multifactorial additive, decontextualized	Non-additive, transactional
Relations	Linear and static	Nonlinear^3^, dynamic and path-dependent^4^

^1^Synergy: spontaneous formation of structural and functional couplings among reciprocally compensating components to achieve task goals [39, 41, 42]

^2^Self-organization: spontaneous order process where some form of overall order arises from local or global interactions between parts of a system, without internal or external programme [43, 44]

^3^Nonlinear means non-proportional: although many CAS' behaviours may for long periods perform in a linear regime (A as independent variable provokes a proportional effect on B over time, i.e., small ΔA = small ΔB or big ΔA = big ΔB), for a certain small change of constraints their dynamics can also become non-proportional (small ΔA = big ΔB)

^4^Path-dependent: CAS’ behaviours are influenced by their past (i.e., history)

**Table 2:** Characteristics of the training process from traditional and complex system-based approaches. Adapted from Balagué et al. [22], with permission

Approach	Traditional	Complex system-based
Role of athletes and coaches	Executers and controllers	Co-designers, co-adapters
Periodization	Preprogrammed	Co-adapted
Monitoring (dominantly)	Objective	Subjective and objective
Measures	Quantitative	Qualitative and quantitative
Monitoring information	Fragmented and decontextualized	Integrated, contextualized

In contrast to complicated machines, CAS’ behaviours cannot be accurately predicted from a particular variable or set of variables. While machine behaviours emerge as a product of their individual component behaviours, in CAS, characterized by an interaction-dominant dynamics, behaviour emerges as a product of the nonlinear integration of personal and environmental influences acting across multiple levels and timescales [45, 46]. A fundamental implication of this inherent and undeniable complexity is that, in the context of complex biological and/or medical contexts, fragmented metrics or even the aggregation of multiple metrics, do not appear to provide meaningful predictive validity [47, 48].

Therefore, some authors have questioned the logic underpinning contemporary trends in sports monitoring [21]. They point out that the empirical assessment of surrogate measures of activity intensity, or isolated measures of physical function, even when blended using integrative algorithms or machine learning techniques, does not provide sufficiently reflective snapshots of current health status and/or performance potential. The prevailing fallacy of completing the puzzle through large quantities of multidimensional (e.g., biomechanical, behavioural, morphological, etc.) athlete-centred objective information lacks theoretical support in uncertain and complex scenarios [49, 50]. Collecting isolated snapshots of a limited number of quantifiable measures, in the absence of a clearly defined and appropriately weighted relational hierarchy of performance priorities, promotes a distorted reality [51, 52]. Importantly, these misleading distortions serve to inappropriately prioritize readily accessible, while neglecting seemingly inaccessible, information [6, 7, 53–55]. A famous quote says ‘not everything that counts can be counted, and not everything that can be counted counts’ [56].

The perpetuation of protocols and procedures developed under the influence of this conventional biomedical depiction of the brain *as a (predominantly) passive, stimulus-driven organ that* waits to receive sensory feedback before processing data and generating responses [57] continues to shape coaches–athlete interactions. The conception of athletes’ as mere executors of coaching instructions does not promote athletes’ awareness, such as quality of attention, depth of understanding and the athletes’ perception of the utility and value of subjective monitoring processes [23]. In addition, this perception dramatically oversimplifies the nature, relevance and influence of interpersonal interactions between coaches, athletes and teams [22, 23]. Through this conceptual lens, the properties which enable CAS to spontaneously, dynamically and adaptively customize behavioural responses to continuously changing context-specific demands are typically either ignored or despised.

As shown in Table 1, acknowledging athletes as CAS drives a reframing of the training process: an appreciation of the mutually entwined integration between biopsychosocial influences, training workloads and training outcomes, while also highlighting existing limitations of conventional athletic monitoring.

In Table 2, the traditional role of coaches is re-envisioned. Instead of fixing objectives and task outcomes, and controlling workloads based on personal opinion blended with monitoring information, through the lens of a CAS-informed philosophy the coach is better perceived as a co-learner, a co-designer and a co-adapter of the training process [23]. A mentor, over time, facilitates the athletes’ journey from dependency to autonomy. Athletes in turn, instead of being mere executers of the coaches’ directives, participate actively in the process, cooperating with the coach in the design and adaptation of training workloads. Although conventional monitoring tools can complement co-designed training processes, the key sources of information come from the athletes’ subjective perceptions.

CAS’ approaches have promoted subjective monitoring [22, 29, 58] but also objective monitoring connected to new methods of analysis or old methods applied to new phenomena [22, 59]. Based on coordination dynamics properties, such methods aim to gain new insights into the changing relationships between groups of variables. Uncontrolled manifold method to capture interpersonal synergies in sport [60], squared coherence to capture cardiorespiratory coupling on acute hypoxia [61], network analysis to capture multilevel organs’ interactions [26, 62] or fractal analysis to capture physiological or kinematic variability of athletes in exhausting exercises [36, 37] may stand as examples. The applicability of this approach, together with the development of adequate technology, should enable in the future a more effective, integrative and realistic encapsulation of CAS’ behaviours. For example, cardiorespiratory coordination variables, extracted by a principal component analysis approach, seem to be more sensitive than common measures (e.g., VO_2max_ or ventilatory thresholds) to reveal specific training adaptations [63, 64], testing manipulations [65], workload accumulation [66] and nutritional interventions [67].

However, at this moment the capacity to quantify, process and instantaneously integrate, interpret and deliver multilevel and multidimensional information may not work if attempted. In contrast, subjective monitoring may provide a practical, efficient and effective means of acquiring continuous high-level actionable information [22, 29, 58].

## Subjective and Objective Sports Monitoring: Advantages and Limitations

**Fig. 1:**
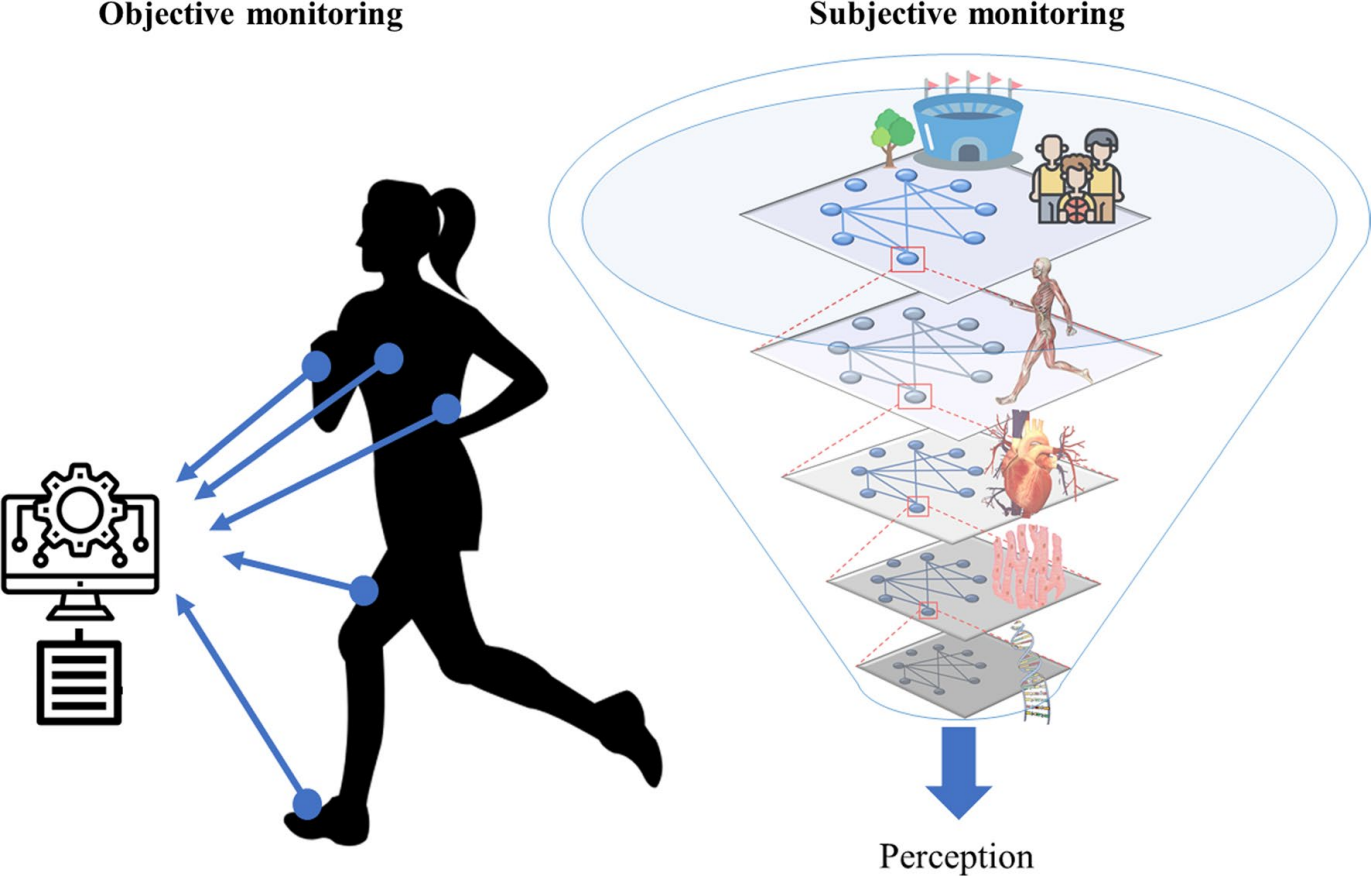
Integrative proposals of objective and subjective monitoring. Objective monitoring: "integration achieved by algorithmic treatment of a collection of independent variables". Subjective monitoring: "integration achieved by experiential dimensional reduction (i.e., information compression) of multilevel organism–environment interactions acting at multiple timescales". Adapted from Balagué et al. [22], with permission

Subjective and objective sports monitoring are distinct in acquiring information and their interpretations do not always agree [18, 68–72]. As illustrated in Fig. 1, objective monitoring commonly fragments the scale of observations to permit the quantitative precision of isolated metrics. Instead of fragmenting, subjective monitoring enables re-composition, integration and dimensional reduction, e.g., compression of psychological, biomechanical and physiological information. Thereby, the generation of subjective perceptions reflects the blended inputs of multiple channels of information, including the organism–environment interaction, highly relevant for health/performance regulation [24, 73]. General health, well-being, recovery, happiness, readiness to train, stress and mood, all stand as examples [74]. Subjective monitoring may also integrate multiple dimensions of awareness, such as proprioceptive [75], kinaesthetic [76], body [77–79], somatic [80], interoceptive [81–84], environmental [85]. Some authors have defined the in situ interactions of these various flavours of awareness, as prospective situational awareness, conceptualizing it under the term of informed awareness [85–87]. In that regard, in comparison with conventional objective monitoring, subjective data collection provides opportunities to capture and integrate online multiple streams of relevant and actionable sensory and perceptual information. The potential of such multidimensional subjective information, dwelling at different timescales [88, 89], relates to the sensitivity for capturing the relevant changes of the organism–environment interaction.

Saw et al. [18] showed through a systematic review that self-reported measures may report acute and chronic training loads with superior sensitivity and consistency than common objective measures (e.g., blood markers, oxygen consumption, heart rate). In fact, subjective monitoring is sensitive to change, not only related to training loads [90–93], but also to overtraining status [18, 94, 95], injuries [96–98], illness [99, 100] or even the rhythms in the earth’s magnetic field [101]. In addition, it may also provide relevant information for predicting individuals’ health-related behaviour and decision-making [102] or performance status [103, 104]. Therefore, some authors suggest that subjectively acquired information can be more effectively used to enhance performance and/or recovery, than information collated via objective monitoring [105–107]. The use of subjective measures is also recommended in bespoke multifactorial models focused on assessing the complex relationship between athlete health, training load, injury and performance [73].

Despite the increasing availability of high-tech-based objective tools, the use of subjective monitoring has recently increased in specific sport contexts [74, 108]. Typically, the RPE remains one of the most popular objective measures of subjective information in sport (e.g., in professional football, [109]) [94, 110, 111]. Other measures to evaluate constructs such as fatigue, pain or internal training loads have also been validated [73, 92, 112–115].

As there is no internal absolute scale for measuring subjective variables, and judgments in psychobiological tasks are context dependent, some researchers have proposed the use of ordinal instead of cardinal scales [116–121]. Such strategies enable capture of the nonlinear dynamics of integrated variables (e.g., perceived exertion, volition states, attention focus) during exercise and may help to anticipate exhaustion and task failure. Effort phases defined by perceived exertion thresholds, informing about the time and workload intensities that can be sustained over time [118], have been detected and correlated with conventional physiological measures of anaerobic threshold [88]. Such ordinal and continuous recording strategies may solve one of the main limitations of subjective monitoring: to precisely transform the complex information of subjective perception(s) in oversimplified numbers [74]. In particular, during exercise this information emerges from the continuous perception of and acting on affordances dwelling at relatively short timescales [86, 122]. Thus, it is possible to increase the monitored sensitivity to qualitative and non-proportional effects of training and/or competition workloads using ordinal recording strategies [88]. Similarly, verbal feedback with the coach serves to improve performance, to monitor progress and to build common trust and buyin of athletes [123].

In addition, subjective monitoring is cheap and simple to implement compared to objective monitoring, which typically requires an investment in technology and qualified human resources [7, 124]. It can be easily interpreted right there and then by athlete or coach, and it is relatively straigh-forward and self-explanatory [125]. It does not depend on good internet connection or some proprietary software, and it also does not pose data risks in terms of storage, breaches, etc., because it can be processed locally. Further, subjective monitoring is applicable to any athlete, regardless of their performance level, sport or economic possibilities. Consequently, investing in subjective monitoring is not only more sustainable but also an economically valid decision.

Finally, a subtle refocussing on subjective monitoring, through the lens of a CAS-informed approach, may serve to enhance athletes’ autonomy, confidence and promote self-regulation capacities [23, 126]. Furthermore, it also serves to enhance athletes’ and coaches’ education and may drive better comprehension of the training process.

**Fig. 2:**
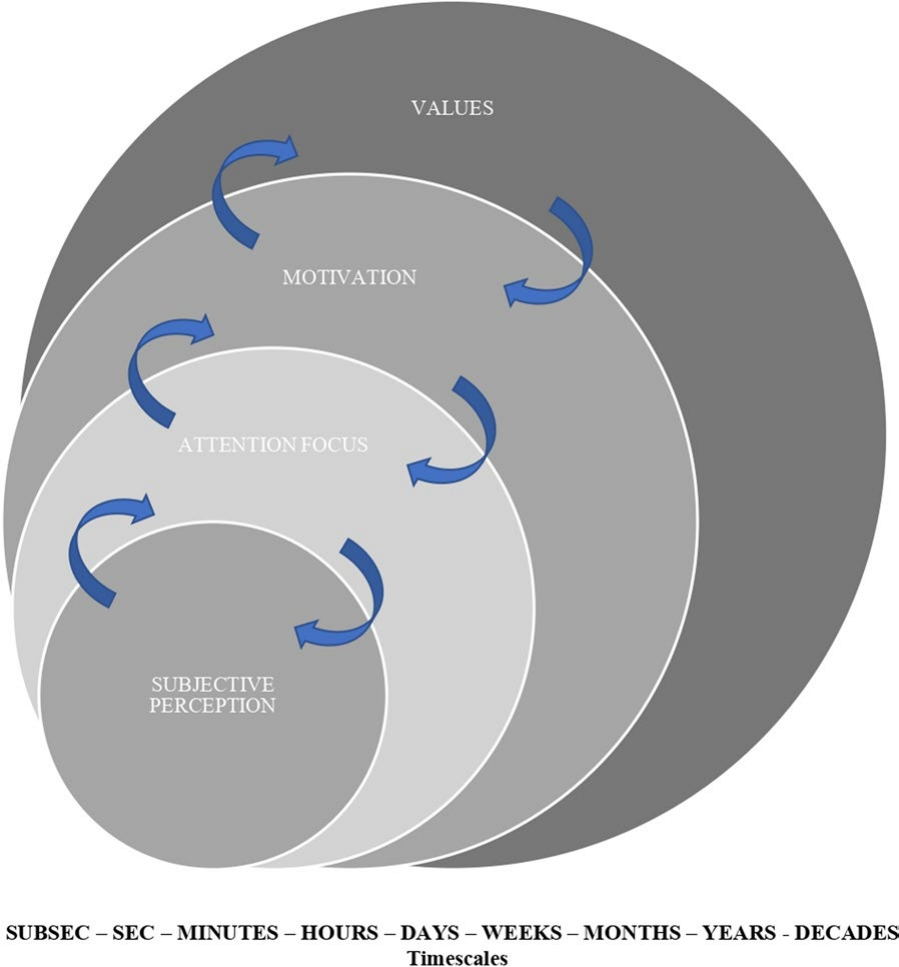
Relations between slow-changing and fast-changing constraints influencing subjective perception. Adapted from Balagué et al. (89), with permission

However, several biases may affect the validity and reliability of subjective measures [124, 127, 128], such as the measure’s sensitivity [73, 129] or individual’s subjectivity [11]. In fact, different personal and environmental constraints influence perception [89]. Values and motivation, stable and slow-changing personal constraints, drive faster changing constraints, such as attentional focus and perception [88, 89] (see Fig. 2). The intervention at the level of more stable constraints (e.g., personal values) has a long-lasting effect on the less stable or faster changing constraints (e.g., perceptions). Thus, an effective application of subjective monitoring depends, to a large extent, on the value athletes and coaches place on subjective monitoring. Accordingly, before using subjective monitoring, athletes and coaches should first be educated on its benefits and acquire enough practical experience. Figure 2 hypothesizes that positive changes in beliefs and values towards self-monitoring may enhance athletes’ awareness, increase their autonomy and self-regulation and, thereby, promote their health and performance. In turn, due to circular causality (see Fig. 2), the enhancement of athletes’ awareness and perception will enhance their attention, motivation and value given to subjective monitoring [80, 81]. This may explain why experienced athletes are often more precise in their subjective reports [124].

The pillars of an effective subjective monitoring are athletes’ self-sufficiency, self-consciousness, autonomy and honesty. Athletes who do not fully understand the rationale underpinning subjective monitoring are unlikely to fully and appropriately engage with the monitoring process and may be more open to providing misleading subjective feedback [73].

In structuring an effective monitoring process, it seems of key importance to distinguish when subjective perceptions are sufficient and when complementary objective information is needed [130]. For instance, the objective information about changes occurring at the molecular level can help to detect dysfunctions when the athlete is feeling well [24] or may alert about possible overtraining effects or microscopic injury processes [131]. It can also promote a feeling of security and trust, which has been effective in spontaneous remissions of serious health problems [132].

Importantly, an exclusive focus on objective monitoring may anaesthetize athletes’ sensitivity to internal and external signals. The common overuse of drugs and ergogenic aids (e.g., anti-inflammatory drugs and hormonal treatments) may also contribute to attenuate body signs, producing a loss of sensitivity in athletes [133]. Further, not only athletes but also coaches may be misled when subordinating their decisions to technology [134]. Thus, a shift of focus is reclaimed to avoid replacing the human perception–action cycle by tech-based feedback and expand the already existing human capacities to promote athletes’ health and performance.

## Challenges for the Future: Educating Athletes’ Awareness

Pain, injuries or disease may increase athletes’ awareness of previously ignored internal and external information [135–138]. However, early education, leading to more informed identification and interpretation of relevant information, may serve to enhance this development [29].

Through subjective monitoring, coaches may promote athletes’ awareness and encourage them to focus their attention on health and performance states. A diversity of training challenges may provide opportunities to generate, experience and practice under different physical, cognitive, affective and environmental conditions, thereby providing brain and body with the richness of sensory experience required to adequately stimulate and promote the development of awareness. In this regard, a variety of movement-based contemplative and therapy approaches, often categorized as mind–body (e.g., Yoga, Qigong, Tai Chi), claim to enhance awareness through movement experiences [78]. In fact, bodily sensory systems are the first to develop and play a fundamental role in the formation of the sense of self, which involves a complex interplay of brain, body and environment information [139–142]. What a living organism senses and perceives is a function of how it moves, and how a living organism moves is a function of what it senses and perceives [143].

Future research should investigate the potential for awareness-promoting strategies and training methods to enhance the value and influence of subjective monitoring systems. Furthermore, the value of educational programmes specifically targeting athletes’ awareness may also provide a productive avenue to promote, in a highly cost-effective manner, self-regulation during training and competition.

## Conclusions

Athlete monitoring technologies are pervasive, rapidly evolving and vigorously marketed. As sports organizations become increasingly inundated with ‘big data’, compiling, integrating and distinguishing between worthwhile and unnecessary informational streams has become a formidable challenge, for researchers and practitioners alike. Mathematical algorithms and artificial intelligence techniques clearly do not adequately capture the self-organized and nonlinear dynamic behaviour of CAS. The presumption that empirical data are superior to information generated by a self-aware, educated and informed athlete is, as highlighted here, fundamentally flawed. Subjective monitoring enables integration, blending and dimensional reduction of the multiple information streams that coalesce to shape the performer–environment system.

The validity and functionality of enhanced subjective monitoring, however, demand that both coaches and athletes are adequately informed and engaged, share a common interpretation of key subjective descriptive terms and share a coherent conceptual understanding of the value of subjective assessments. Positive changes in beliefs about self-monitoring may enhance athletes’ awareness, increase engagement, autonomy and self-regulation, and thereby, promote health and performance. This is not an argument to eliminate objective assessments, but to restore badly needed balance and to realign monitoring convention with theory and evidence. Critically, in the future, there is a need to evolve more discerning methods for creatively capturing, interpreting and presenting subjective athlete-generated information. Similarly, there is a clear need to provide specific education programmes to improve both athletes’ awareness and coaches’ understanding of the potential of subjective monitoring and training prescription to enhance both performance and health outcomes.

## Data Availability

Not applicable.

## Abbreviations

ACWR: Acute: chronic workload ratio
CAS: Complex adaptive systems
RPE: Rating of perceived exertion
VO_2max_: Maximal oxygen consumption
