# Correction: Integrative Proposals of Sports Monitoring: Subjective Outperforms Objective Monitoring

**DOI:** 10.1186/s40798-022-00460-9

**Published:** 2022-05-06

**Authors:** Lluc Montull, Agne Slapšinskaitė-Dackevičienė, John Kiely, Robert Hristovski, Natàlia Balagué

**Affiliations:** 1 Complex Systems in Sport Research Group, Institut Nacional d’Educació Física de Catalunya (INEFC), Universitat de Barcelona, Barcelona, Spain; 2 University School of Health and Sport, University of Girona, Girona, Spain; 3 Department of Sports Medicine, Faculty of Nursing and Faculty of Public Health, Health Research Institute, Medical Academy, LSMU, Kaunas, Lithuania; 4 Institute of Coaching and Performance, School of Sport and Wellbeing, University of Central Lancashire, Preston, PR1 2HE UK; 5 Complex Systems in Sport Research Group, Faculty of Physical Education, Sport and Health, Ss. Cyril and Methodius University in Skopje, Skopje, Macedonia

## Correction to: Sports Medicine—Open (2022) 8:41 https://doi.org/10.1186/s40798-022-00432-z

The original article contained a minor error in Affiliation #3 which has been updated to the following:

Department of Sports Medicine, Faculty of Nursing and Faculty of Public Health, Health Research Institute, Medical Academy, LSMU, Kaunas, Lithuania.

